# Dental age assessment in 6- to 14-year old German children: comparison of Cameriere and Demirjian methods

**DOI:** 10.1186/s12903-016-0315-8

**Published:** 2016-11-08

**Authors:** Thomas Gerhard Wolf, Benjamín Briseño-Marroquín, Angelika Callaway, Michael Patyna, Victor Thomas Müller, Ines Willershausen, Vicky Ehlers, Brita Willershausen

**Affiliations:** Department of Operative Dentistry, University Medical Center Mainz, Augustusplatz 2, D-55131 Mainz, Germany

**Keywords:** Accuracy, Cameriere’s method, Dental age estimation, Demirjian’s method, Forensic dentistry, German population

## Abstract

**Background:**

The aim of the study was to compare two frequently used dental age estimation methods for accuracy.

**Methods:**

A total of 479 panoramic radiographs in age groups 6–14 years from a German population were evaluated. The dental age of 268 boys and 211 girls was assessed by means of the method of Demirjian (1973) and Cameriere (2006) and compared with their actual chronological age.

**Results:**

Demirjan’s method showed an overestimation of dental age compared to chronological age in all age groups for boys (mean difference −0.16, *p* = 0.010, range −0.35 to 0.09), age group 9 showed an underestimation. Using the same method for girls (mean difference −0.18, *p* = 0.008, range −0.45 to 0.13), an overestimation could also be shown in all age groups except for age groups 8 and 13. Results for Cameriere’s method showed for boys (mean difference 0.07, *p* = 0.314, range −1.38 to 3.83) in age groups 6 to 11 an overestimation, but in age groups 12 to14 an underestimation. The results for girls (mean difference 0.08, *p* = 0.480, range −1.55 to 4.51) showed an overestimation for age groups from 6 to 10, and an underestimation in age groups 11 to 14.

**Conclusions:**

The comparison shows an advantage of Demirjian’s method for both genders. While Cameriere’s method showed a higher inaccuracy in all age groups, Demirjian’s method showed more appropriate results for dental age estimation of the investigated German population. To avoid errors in forensic age estimation and to prevent misidentifications for defendants in criminal processes, further studies of more precise methods for age estimation for the German population are required.

## Background

Dental age estimation plays an essential role in forensics, anthropology and bioarchaeology [1]. Both for living and dead individuals, a precise age estimation is required, especially in children and young adolescents [2]. In living individuals, judicial challenges must be solved when valid identification documents are lacking [2, 3]. While cementum or dentine can be studied in dead bodies, the focus for the living is on the clinical situation and radiographs [4]. Root development, periodontium, ratio between pulp and tooth or tooth and root of permanent teeth can be measured radiologically. Methods such as evaluation of tooth morphology [5, 6], morphology of the primary and permanent dentition [7], degree of ossification of skeletal structures [8], evaluation of biochemical findings in the dental hard tissue and the investigation of age-dependent changes in the human genome [9] show different results regarding accuracy and possible means of application. Human dentition and human maturation processes also offer good assessability due to the high degree of independence from environmental factors and systemic diseases [2, 3]. Most accurate methods of dental age estimation in children are based on the radiologically observed tooth development of the permanent teeth [3]. For forensic dentistry the method must obtain precise, reliable and comparable results when used by different investigators and the results must come close to the real age [10]. Due to differences in dental maturation process in geographic and ethnic origin and discrepancies of results of radiographic methods used for dental age estimation [1], there is a lack of data concerning the German population. This is the first study, that addresses the comparison of both methods of Cameriere and Demirjian of a German sample. Although the method of Cameriere [11], that is based on the relationship between age and measurement of the open apices in teeth with a formula for European populations, and Demirjan’s method [7] investigating seven teeth on the left side of the mandible and based on a French-Canadian dataset, there is a need in investigating the two often used methods for accuracy and reliability in a German population. Therefore, the aim of the recent study was to compare the two most frequently used radiographical methods of dental age estimation, the method of Demirjian [7] with the method of Cameriere [11] in a population of German children aged 6–14, to evaluate both techniques, and to demonstrate the differences.

## Methods

### Subjects

For this retrospective investigation, a total of 479 panoramic radiographs were assessed. The orthopantomograms of 268 male and 211 female 6- to 14-year old patients were investigated. A balanced group of subjects was necessary to evaluate differences between both techniques. Therefore, the age groups of 5 (4.6–5.5) and 15 (14.6–15.5) were excluded due to an unequal gender distribution. A total of 479 subjects (268 male, 211 female) were finally included (Table 1). Inclusion criteria were a maximum of one aplastic or missing tooth and only orthoradially depicted teeth.

**Table 1:** Distribution of the children (*n* = 479) according to age in years (minimum-maximum) and gender

Age	Boys	Girls	Total
6 (5.6–6.5)	18	11	29
7 (6.6–7.5)	15	16	31
8 (7.6–8.5)	27	26	53
9 (8.6–9.5)	38	43	81
10 (9.6–10.5)	48	30	78
11 (10.6–11.5)	41	26	67
12 (11.6–12.5)	31	29	60
13 (12.6–13.5)	34	18	52
14 (13.6–14.5)	16	12	28
Total	268	211	479

*p* > 0.05 for all age groups

### Methods

Panoramic radiographs were taken in the period of 1986 to 2005 at the Department of Dental Radiology of the University Medical Center of the Johannes Gutenberg University Mainz, Germany. The radiographic devices Orthophos 3 (Siemens, Germany, 208/230 V, 9 A), Orthophos CD (Siemens, Germany, 90 kV; 12 mA), and, until 1994, Cronex dental films (Dupont, Germany) were used. The chronological age (CA) for each subject was calculated by subtracting the date of the panoramic radiograph from the date of birth. After converting to the decimal age, for example, patients with ages ranging from 5.6 to 6.5 were designated as the 6-year group. The radiographs used were retrieved from the archive according to ethical guidelines; no radiographs were taken solely for this investigation.

For application of the Demirjian technique [7], seven permanent teeth of the left mandible were investigated. Each tooth was classified according to the maturation score according to Demirjian’s method. In 1973 the authors introduced a system of eight stages of maturation or development (A-H) of the teeth evaluated in the panoramic radiograph. The different stages were identified with the help of Demirjian’s maturation stage charts using the overall score of the different point values. After addition of the seven scores, the total score was assigned in the Demirjian table [7].

For application of Cameriere’s technique [11] seven permanent teeth of the right mandible were evaluated. The number of teeth with complete root development and apical ends of the roots completely closed (N_0_) was calculated. Teeth with incomplete root development and with open apices were considered. For teeth with one root, the distance between the inner sides of the open apex was measured. For teeth with two roots, the sum of the distances between the inner sides of the two open apices was analyzed. The formula of Cameriere is: Age = 8.791 + 0.375 g + 1.631 × 5 + 0.674 N_0_–1.034 s–0.176 s ⋅ N_0._ G is a variable equal to 1 for boys and 0 for girls [11].

### Statistical analysis

Statistical analysis was carried out in cooperation with the Institute of Medical Biostatistics, Epidemiology and Informatics of the University Medical Center Mainz. The data of all subjects were analyzed with SPSS 22.0 (SPSS Inc., Chicago, IL). All datasets were evaluated by a single observer (VM). For both methods, data for the variables sex, date of radiograph, date of birth and chronological age were collected. Each patient’s date of birth and the date of the radiograph were noted and the chronological age of the subjects at the time of the recording was calculated exactly. The differences between chronological and dental age were analyzed using the Wilcoxon signed rank test for paired samples with a non-normal distribution. A *p*-value of < 0.05 was considered as statistically significant.

## Results

**Fig. 1:**
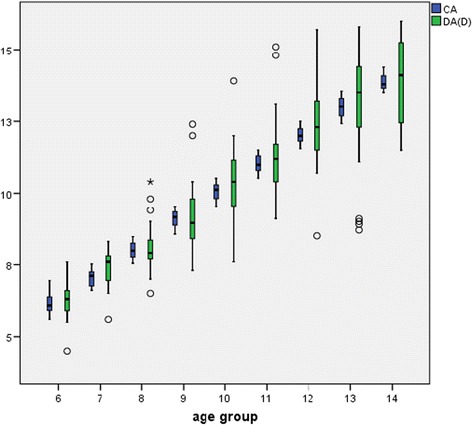
Distribution of chronological age (CA) and dental age (Demirjian scores) DA (D) for boys in different age groups (6–14 years). Shows a linear relation with an overestimation in all age groups, except for age group “9 years”

**Fig. 2:**
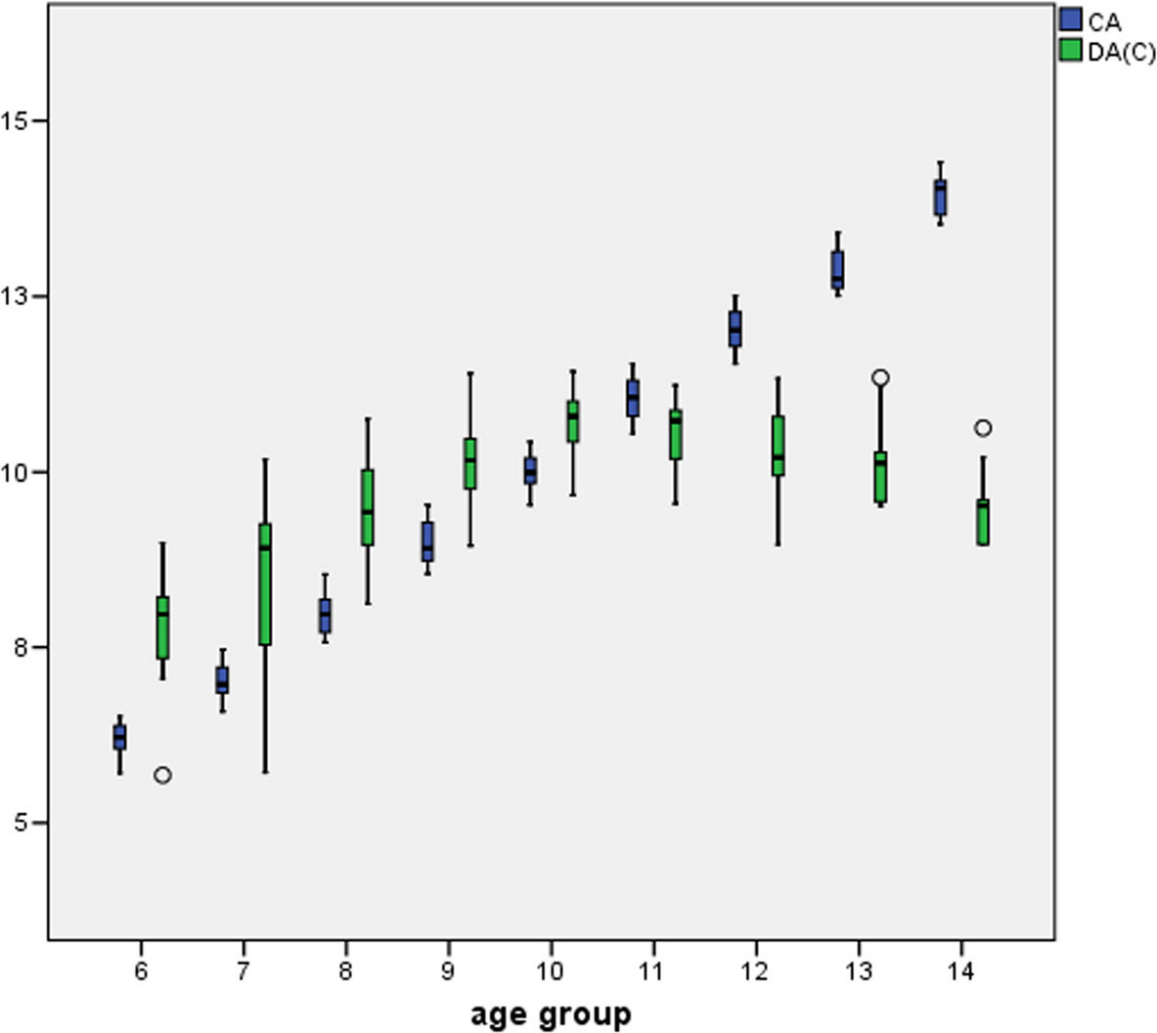
Distribution of chronological age (CA) and dental age (Cameriere scores) DA (C) for girls in different age groups (6–14 years). Shows an overestimation in age groups 6 to 10 and an underestimation in age groups 11 to 14

The distribution of the 479 subjects (268 boys and 211 girls) by age group and gender is shown in Table 1. The distribution of chronological age (CA) and dental age (DA) for boys shown in Fig. 1 in different age groups for Demirjian scores is representative for both genders. In Fig. 2 the distribution of chronological age (CA) and dental age (DA) are shown; these are also representative for both genders for Cameriere scores.

### Demirjian’s scores

The mean dental age as well as the mean difference between chronological age (CA) and dental age (DA) in years using the Demirjian method with *p*-values for the different age groups and divided by gender is shown in Table 2. Demirjan’s method showed an overestimation in age groups for boys, except for age group 9 showed an underestimation. Using the same method for girls, an overestimation could also be shown in all age groups, except for age groups 8 and 13.

**Table 2:** Mean dental age (±SD), difference between chronological age (CA) and dental age (DA) in years (mean ± SD, median, 1. and 3. quartile and interquartile range) using the Demirjian method

	Boys							Girls						
Age Group (years)	Mean Dental Age (± SD)	Mean Difference CA-DA	Median	Q1	Q3	IQR	*p*-value	Mean Dental Age (± SD)	Mean Difference CA-DA	Median	Q1	Q3	IQR	*p*-value
6	6.32 (±0.72)	−0.20	−0.26	−0.53	0.14	0.67	0.078	6.57 (±0.50)	−0.39	−0.37	−0.71	−0.09	−0.80	0.008*
7	7.36 (±0.73)	−0.34	−0.48	−0.66	−0.03	−0.69	0.020*	7.16 (±0.71)	−0.13	−0.28	−0.36	0.03	0.39	0.079
8	8.11 (±0.82)	−0.10	−0.09	−0.53	0.34	0.87	0.866	7.84 (±0.50)	0.12	0,17	−0.08	0.47	0.55	0.142
9	9.00 (±1.15)	0.09	0.26	−0.51	0.98	1.49	0.277	9.03 (±1.02)	−0.01	−0.05	−0.68	0.82	1.50	0.828
10	10.32 (±1.21)	−0.27	−0.28	−1.13	0.39	1.52	0.058	10.41 (±1.44)	−0.40	−0,09	−1.56	0.54	2.10	0.289
11	11.20 (±1.35)	−0.17	−0.24	−0.74	0.71	1.45	0.364	11.48 (±1.22)	−0.43	−0.41	−0.91	0.51	1.42	0.131
12	12.35 (±1.40)	−0.35	−0.24	−1.34	0.35	1.69	0.153	12.30 (±1.57)	−0.29	−0.71	−1.22	0.38	1.60	0.076
13	13.07 (±1.96)	−0.07	−0.48	−1.32	0.91	2.23	0.379	12.73 (±1.51)	0.13	−0.14	−0.97	0.88	1.85	0.632
14	13.90 (±1.54)	−0.05	−0.10	−1.45	1.45	2.90	0.796	14.42 (±0.97)	−0.45	−0.38	−1.03	0.14	1.17	0.099
Total	10.41 (±2.51)	−0.16	−0.21	−0.09	0.58	0.67	0.010*	10.18 (±2.46)	−0.18	−0.22	−0.79	0.47	1.26	0.008*

*p < 0.05, Q1 = 1. quartile, Q3 = 3. quartile, IQR = interquartile range

The mean dental age as well as the mean difference between chronological age (CA) and dental age (DA) in years using the Cameriere method with *p*-values for the different age groups and divided by gender is shown in Table 3. Results for boys showed an overestimation in age groups 6 to 11, age groups 12 to14 an underestimation. The results for girls showed an overestimation for age groups from 6 to 10, underestimation in age groups from 11 to 14.

**Table 3:** Mean dental age (±SD), difference between chronological age (CA) and dental age (DA) in years (mean ± SD, median, 1. and 3. quartile and interquartile range), using the Cameriere method

	Boys							Girls						
Age Group (years)	Mean Dental Age (± SD)	Mean Difference CA-DA	Median	Q1	Q3	IQR	*p*-value	Mean Dental Age (± SD)	Mean Difference CA-DA	Median	Q1	Q3	IQR	*p*-value
6	7.16 (±1.41)	−1.04	−1.15	−1.81	−0.15	−1.96	0.004*	7.73 (±0.92)	−1.55	−1.75	−2.18	−1.03	−3.21	0.004*
7	8.40 (±1.34)	−1.38	−1.82	−2.09	−0.13	−2.22	0.004*	8.52 (±1.18)	−1.49	−1.76	−2.38	−0.63	−3.01	0.001*
8	9.32 (±0.90)	−1.31	−1.44	−1.81	−0.83	−0.98	<0.001*	9.48 (±0.68)	−1.52	−1.70	−1.85	−1.03	−2.88	<0.001*
9	10.15 (±0.76)	−1.06	−1.08	−1.53	0.63	2.16	<0.001*	10.17 (±0.52)	−1.16	−1.17	−1.46	−0.83	−2.29	<0.001*
10	10.96 (±0.48)	−0.91	−0.97	−1.19	−0.62	−1.81	<0.001*	10.67 (±0.46)	−0.67	−0.72	−1.07	−0.27	−1.34	<0.001*
11	11.05 (±0.50)	−0.02	−0.08	−0.49	0.31	0.8	0.489	10.55 (±0.48)	0.50	0.40	−0.07	0.95	1.02	0.001*
12	11.01 (±0.65)	0.99	0.86	0.43	1.57	1.14	<0.001*	10.26 (±0.68)	1.75	1.87	0.99	2.25	1.26	<0.001*
13	10.49 (±0.77)	2.51	2.69	1.63	3.27	1.64	<0.001*	10.14 (±0.61)	2.72	2.90	2.29	3.16	0.87	<0.001*
14	10.02 (±0.75)	3.83	3.88	3.32	4.64	1.32	<0.001*	9.46 (±0.54)	4.51	4.65	3.97	5.14	1.17	0.002*
Total	10.19 (±1.34)	0.07	−0.41	−1.20	1.06	2.80	0.314	9.92 (±1.01)	0.08	−0.50	−1.29	1.26	2.55	0.480

*p < 0.05, Q1 = 1. quartile, Q3 = 3. quartile, IQR = interquartile range

**Fig. 3:**
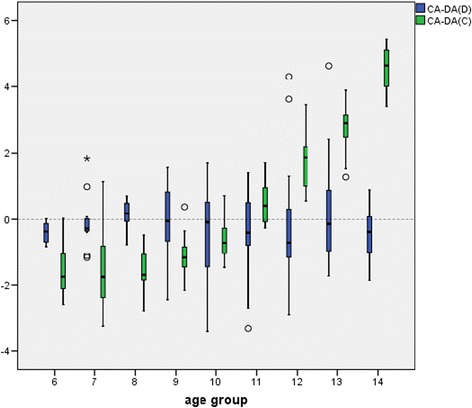
Differences between chronological age and dental age estimated for girls. CA-DA(D) = chronological age – dental age (Demirjian scores); CA-DA(C) = chronological age – dental age (Cameriere scores). There was a linear accordance of the results of Demirjan’s method in the age groups of 6 until 14 years. The Cameriere scores show an increasing slope from 6 to 14 years with an overestimation in age groups 6–10 and an underestimation in age group 11 to 14. The inaccuracy increases from 12 to 14

Differences between chronological age and dental age estimated for girls can be seen in Fig. 3. A linear accordance of the results of Demirjian’s method in the age groups of 6 until 14 years can be observed. The Cameriere scores showed an increasing slope from 6 to 14 years with an overestimation in age groups 6–10 and an underestimation in age group 11–14. The inaccuracy increases clearly from 12 to 14.

**Fig. 4:**
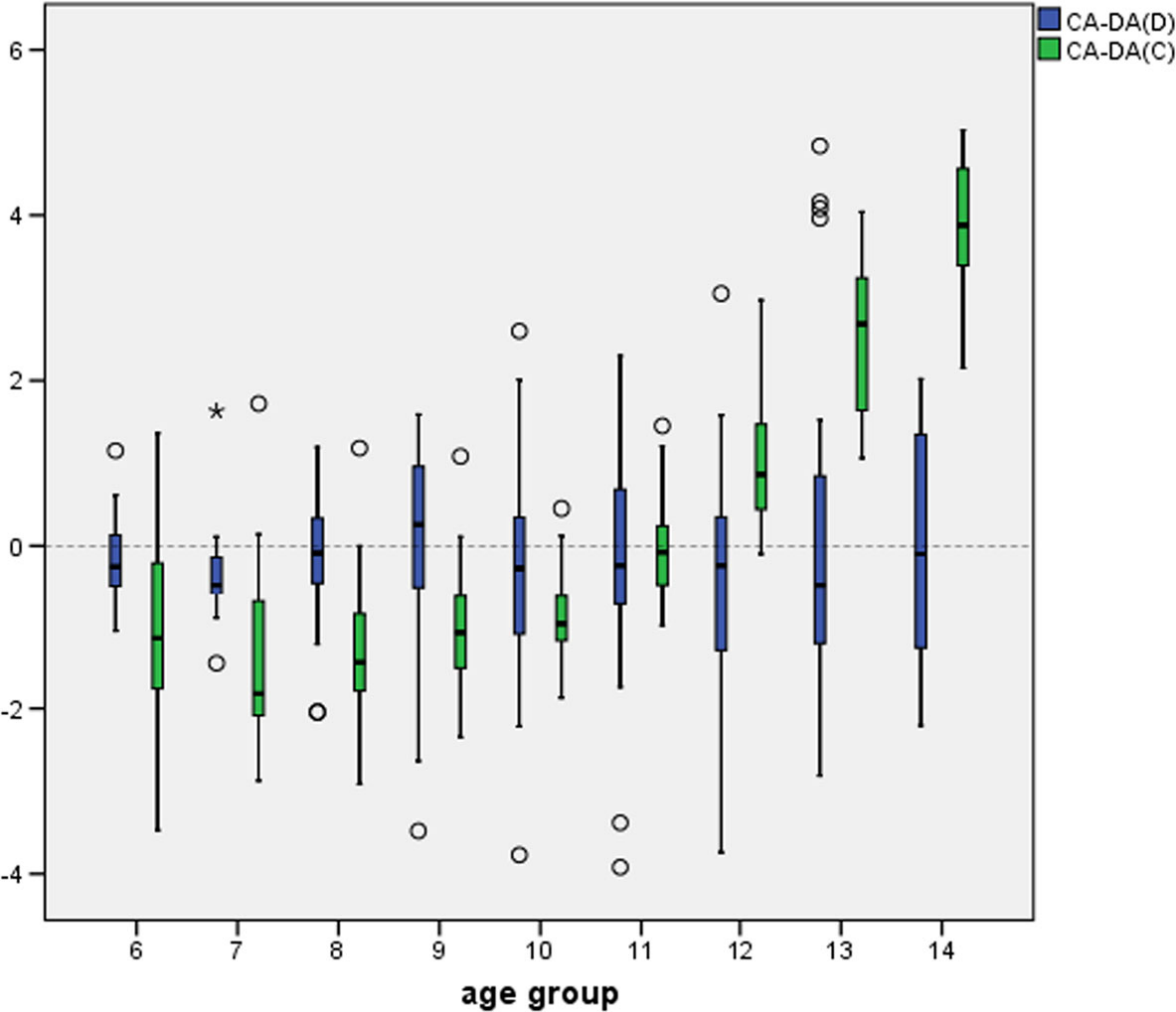
Differences between chronological age and dental age estimated for boys. CA-DA(D) = chronological age – dental age (Demirjian scores); CA-DA(C) = chronological age – dental age (Cameriere scores). The comparison of the two methods shows an advantage of the Demirjian method for both genders

Differences between chronological age and dental age estimated for boys (Fig. 4) showed similar results to that for girls for both methods.

The comparison of the two methods shows an advantage of the Demirjian method for both genders.

## Discussion

The estimation of age in children is an important challenge in orthodontic treatment, pediatric endocrinology and forensic medicine and dentistry [11]. Moreover, in many European countries the number of foreigners, immigrants and refugees without valid identification documents or date of birth is rising [12]. This development leads to an upcoming opportunity for diagnostic investigation of the assessment of age in living individuals. Considering that numerous diagnostic methods exist [13, 14], only few of them seem to be appropriate for usage in forensic contexts in living individuals, taking ethical and medicolegal aspects into account [12]. Due to the fact that environmental factors in dental age estimation methods are fewer than in skeletal methods with an influence on the variability of bone maturation [11, 15], alternative methods based on gene-controlled dental development appear to be clinically suitable for age estimation [16, 17]. The methods used must be suitable for obtaining reproducible, precise and reliable results [1]. For the assessment of mineralization within acceptable error limits, several methods [7, 11] have been realized in the past [18]. Radiographic non-invasive techniques are useful both for living and dead, in forensics and archaeology [19]. In this study, the investigated radiographic and commonly used methods of Cameriere [11] and Demirjian [7] were evaluated with respect to their accuracy and compared with each other, dividing the German study population by age groups and sex.

A great variability in the dental maturation process in geographic and ethnic origin can be seen around the world [20–22]. For the German population we found in literature only two papers containing data concerning dental age estimation of both methods investigated [23, 24]. While Demirjian’s method is based on a French-Canadian data collection and describes results in the study of Frucht et al. [20], the data set of Cameriere et al. [24] offers results by measurement of open apices in teeth in a European formula, where German children were included. The authors [24] already claimed that studies are needed comparing the reliability of their method [11] with other methods for age estimation, in particular among others with Demirjian’s method [7].

Overestimation of age has been reported frequently [22], but underestimations have also been reported [18, 25]. Results from the present study (*n* = 419 [211 f/268 m], 6–14 years old, −0.18 f and −0.16 m) compared to other countries located geographically in Europe are close to those of Maber et al. [3], United Kingdom *n* = 946 [491 f/455 m], 3–17 years old, −0.23 f and −0.25 m) and Nykänen et al. [21], Norway *n* = 300 [150 f/150 m], 5.5–12.5 years old, −0,30 f and −0.20 m with updated dataset [26]. Both studies reported an overestimation of dental age in boys and girls. A slightly higher discrepancy in results, but also overestimation, were determined by Willems [1] (*n* = 2116 [1029 f/1087 m], 3–18 years old, −0.7 f and −0.4 m), Foti et al. [27] (*n* = 100 [49 f/51 m], 6–21 years old, −0.92 f and −0.82 m with table value calculation), Leurs et al. [28] (*n* = 451 [226 f/225 m], 3–17 years old, −0.60 f/-0.40 m), Cruz-Landeira [18], *n* = 308 [151 f/157 m], 2–18, −0.88 f and −0.76 m) and Hagg & Matsson [29] (*n* = 300 [150 f/150 m], 3.5-12.5, −0.61 f and −0.51 m with converted value and table value calculation). Discrepancies can be explained by differences in geography, countries and places, investigated. Different ethnicities, sample sizes and age groups as well as statistical adjustments, sometimes not available, are possible explanations for the differences.

In the present study, the dental age assessment by method suggested by Cameriere showed a slight difference for boys (0.07, range −1.38 to 3.83) and for girls (0.08, range −1.55 to 4.51). These findings are in concordance with the results of Gulsahi et al. for boys (−0.47, range −3.70 – 4.06) and for girls (−0.24, range −2.74 to 3.29) [30]. But the fact that differences between dental and chronological ages decrease with increasing chronologic age [30] could not be affirmed in the present study; starting from 12 to 14 years we observed increased inaccuracy. The circumstance that dental age assessment for girls by the method of Cameriere is more accurate than for boys [30] could not be confirmed either. We agree with the results of Cameriere et al. [15], who reported a slight underestimation of the real age of children investigated, but disagree that 90 % of the absolute value of residual errors obtained was less than one year. The results from another study [31] showed similar outcomes with an underestimation by mean age by 0.02 years for boys and an overestimation by mean age by 0.29 years for girls. After examination with two other techniques for dental age estimation, Galic et al. concluded that the Cameriere method was the most accurate method for 6–13 year old Bosnian-Herzegovian children [31]. We cannot agree with this outcome in light of the findings of the present study.

The results form the present study are in contrast with the findings of Carneiro et al. [32], who attested that Demirjian’s method was an unsuitable method according to their Portuguese study sample with a systematic bias and consistent inaccuracy. We cannot concur with the decision to abandon Demirjian’s method for forensic age estimation. Quite the opposite, we recommend further research, as do other authors [33], to obtain more accurate results examining sample groups of different ethnic and geographic origin.

Although with increasing age, gender differences vary [34], we could not find the expected significant sex differences reported, that girls mature faster than boys [34, 35].

Findings from the present study showed that the method introduced by Demirjian [7] appears to be more accurate in the studied sample than the method of Cameriere [11]. Gender seems to have had no influence on the results. The method of Demirjian [7] is well suited for dental age estimation of young German individuals 6–14 years old.

## Conclusions

Demirjian’s method is suitable for estimating the age of boys and girls of 6–14 years in a German population. Demirjian’s method is superior to Cameriere’s method for dental age estimation of 6–14 years old children in a German population.

## Abbreviations

C: Cameriere
CA: Chronological age
D: Demirjian
DA: Dental age
f: female
m: male
n: quantity
